# Formulating a Need-Based Faculty Development Model for Medical Schools in Indonesia

**DOI:** 10.21315/mjms2019.26.6.9

**Published:** 2019-12-30

**Authors:** Riry Ambarsarie, Rita Mustika, Diantha Soemantri

**Affiliations:** 1 Department of Medical Education, Faculty of Medicine, University of Bengkulu, Kandang Limun, Bengkulu, Indonesia; 2 Department of Medical Education, Faculty of Medicine, Universitas Indonesia, Jakarta, Indonesia

**Keywords:** faculty development, model, medical education, medical teacher

## Abstract

**Background:**

The focus of medical schools in developing countries is on fulfilling a quantity of faculty members. A faculty development model will help formulate programmes that accommodate faculty members’ needs as well as institutional demands. This study aims to formulate a faculty development model relevant for medical schools in developing countries, specifically Indonesia.

**Methods:**

This is a qualitative study with a phenomenological approach. It starts with a literature review using large databases, followed by interviews with 10 representative experts from medical schools in Indonesia.

**Results:**

Based on the 10 studies retrieved, several components of faculty development were identified as the basis for the model. Ten experts gave input for the model. Components of the model can be grouped into: (i) content, which is materials that need to be delivered; (ii) process components, which depict aspects related to the preparation, execution and evaluation of sustainable faculty development; and (iii) components in the educational system that affect faculty development implementation.

**Conclusion:**

A comprehensive review and development process has likely made this faculty development model suitable for medical schools in Indonesia. Breaking the model into components may help medical schools to prioritise certain aspects related to faculty development programmes.

## Introduction

A faculty is the most important resource in a higher education institution because improvement of educational quality is mostly influenced by the quality of the faculty. In other words, one factor that could improve the quality of a higher education institution is the systematic implementation of a faculty development programme (1). Faculty development is a formal or informal activity (2) that is performed to improve the required knowledge and skills of faculty in their performance and to support the various roles of faculty in education, research, service and administration. This is done in continuous stages and can be measured (3) to improve the quality and work culture of an institution (4).

The faculty development model is a management system for a comprehensive development programme that includes self-development (attitude), instructional development (process) and organisational development (structure) (5). The faculty development model helps identify a programme that can accommodate both the faculty’s needs and academic and institutional demands (1). In formulating the faculty development model, several aspects need to be considered: the theoretical framework used; the focus of faculty development and its role in the curriculum and organisation; the integration of learning into a workplace; and the expectations of the entire academic community (6).

The design of a faculty development model depends on the context in which the model will be used. One of the problems in faculty development in developing countries, especially in Southeast Asia, is the different learning culture of medical schools compared to those in Western countries (7). These differences, such as characteristics of the faculty and institutional culture, make it difficult for many countries in Southeast Asia to adopt faculty development models formulated in developed countries. Various institutions in Southeast Asian countries have tried to implement faculty development programmes, but the lack of experience and funding concerns were the main obstacles in building a comprehensive programme (8).

A ‘developing country’ is a term generally used to define a country with a low level of welfare. Indonesia is a developing country in Southeast Asia. The low per capita income of Indonesian citizens affects the higher education faculty hiring process. Sometimes the effort to recruit faculty members is only aimed at filling the number of vacancies without considering the competency of those members (9). This certainly impacts the quality of education in Indonesia.

Indonesia has 83 medical schools with various accreditation levels (10). The national accrediting agency requires medical schools to develop a system of staff recruitment, development, monitoring and evaluation (11). Therefore, it can be assumed that medical schools with the highest level of accreditation are likely to have a more comprehensive and systematic faculty development programme. The fact that only 27.7% of medical schools have obtained the highest level of accreditation (10) indicates that most medical schools are still struggling to provide high quality medical education, a main cause being the lack of high quality faculty members. One of the means to produce high quality faculty members is through the implementation of a faculty development model. This research aims to formulate a faculty development model that is suitable to the needs of medical schools in Indonesia based on a literature review and exploration of experts’ perceptions through interviews.

## Materials and Methods

This study used a qualitative method with a phenomenological approach. This approach is appropriate to explore the essence of stakeholders’ experiences in faculty development, so that a better understanding of these phenomena in our context can be obtained (12). Data collection was conducted in two steps: literature review and in-depth interviews.

### Literature Review

The review was conducted systematically to identify faculty development models that currently exist in the literature, especially in medical schools. The steps for the literature review were as follows: (i) formulate the search strategy, (ii) identify relevant studies, (iii) analyse the data and (iv) report the results.

## Formulating the Search Strategy

The search process was conducted using two large databases, PubMed and ERIC and applying three sets of combined keywords: (i) faculty development/faculty training/staff development, (ii) medical education/medical school/higher education and (iii) model/framework/programmes. The search focused on English-language journals published in the last 10 years.

## Identifying Relevant Studies

Inclusion criteria in this study were research articles using primary data with various empirical elements; research articles focused on faculty development in medical schools, higher education or other health profession educational setting; and accessible full-text versions. The exclusion criteria were research articles written in languages other than English and no access to the full-text version of the article.

## Analysing the Data and Reporting the Results

The primary researcher (RA) read all titles of the articles collected and eliminated articles with similar titles and unrelated subjects. Then, RA filtered the articles based on information in the abstract. The full text of the remaining articles was reviewed by RA and a co-researcher (DS) to obtain the final articles that fulfilled the inclusion criteria. Data analysis started with reduction, including summarising, selecting and focusing on important matters and removing unnecessary information, then applying codes to certain aspects. Data were extracted from selected articles using Steinert’s framework (13). The results were presented in the form of diagrams, tables and narrative texts.

### In-depth Interviews with Experts

The in-depth interviews involved experts in faculty development from several medical schools in Indonesia. The criteria for selecting medical schools were regional representation, type of institution (public or private) and the school’s level of accreditation. Experts interviewed were medical education experts with either a formal master’s degrees in medical education or a doctoral degree in medical education in progress, along with work experience as a faculty member for at least five years.

Interview questions were developed based on the literature on faculty development and the initial concept of the faculty development model based on the literature review results. Each interview was recorded and lasted around 60 min. Afterwards, an interview report was prepared for the interview subject’s approval of the contents. The results were transcribed verbatim and analysed using thematic analysis (14, 15).

This study obtained ethical clearance from the university’s Medical and Health Research Ethics Committee. Participation in this research was voluntary, and participants provided their written consent prior to the interview.

## Results

### Literature Review

**Figure 1:**
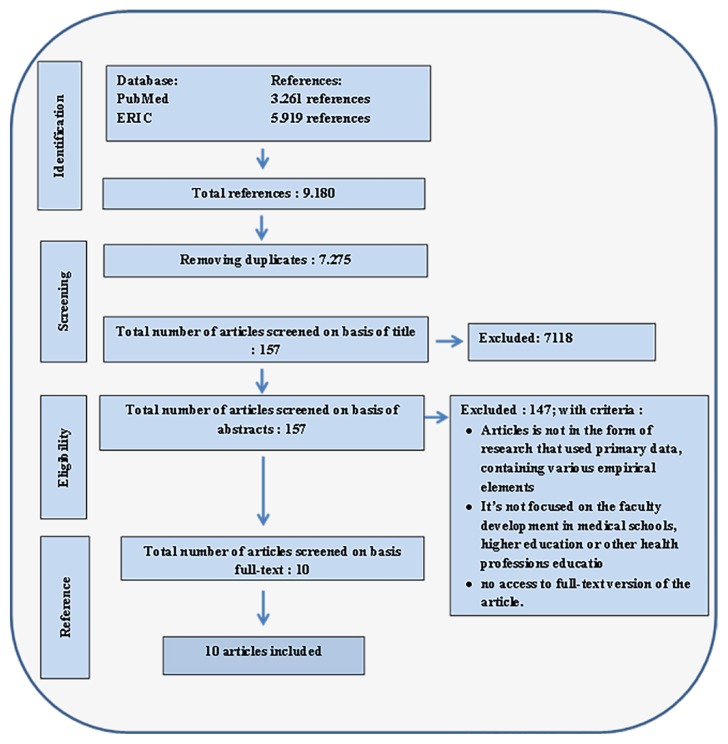
Diagram depicting the flow of inclusion/exclusion criteria

The search process resulted in 9,180 titles from two databases. Following the application of inclusion and exclusion criteria (Figure 1), 10 articles were selected to be reviewed. Table 1 summarises the data extracted from each article.

Most articles described the definition and objectives of a faculty development programme. Four articles specifically discussed a faculty development programme with specific contexts, such as programmes in higher education, programmes for clinical faculty members and programmes for faculty members of medical schools in general (8, 16, 17, 18).

Eight studies implied an extended definition of faculty development that was not only developing teaching skills but also personal and professional skills as a teacher, clinician, researcher and administrator relevant to the vision and mission of the institution. Most studies stated that to prepare comprehensive and effective faculty development, a needs analysis is required, for example, in relation to teaching clinical skills, the latest teaching and assessing methods (19), technology in teaching (20, 21), appropriate teaching methods for current student generation and online teaching (22, 23, 24). Faculty development also needed training on publications, managing time for research and writing a manuscript (8, 18, 20). Two studies (20, 22) described other skills: management and leadership skills.

Approaches and types of faculty development activities can be divided into three categories: (i) independent learning, which was conducted in nearly 50% of the whole faculty development activities, especially in improving pedagogic skills (7, 19, 20, 23, 25); (ii) formal professional development programmes dominated by continuing education programmes, seminars and workshops (19, 20, 24); and (iii) organisational development strategies to improve organisation quality, such as curriculum development towards online learning, development of faculty development programmes across institutions, online-based faculty development and innovations in professional development models (18, 23, 26).

Topics related to pedagogic competencies, such as teaching skills, were still the main choice in faculty development, especially those related to current student generation, class preparation, large class teaching, counseling and communications (8, 19, 21, 22). Other topics were the application of technology and information in the educational process, such as the use of technology in teaching, the implementation of technology-based faculty development programmes and challenges in online-based learning (21, 23, 24).

Two studies specifically explored the approaches used in evaluating faculty development activities. One was through participants’ satisfaction surveys, and the other was based on performance changes as described by evaluation reports and interviews of fellow faculty and students (24, 25).

Based on the findings from the literature review, we developed a faculty development model consisting of components such as instructional, organisational, leadership and professional development influenced by medical schools’ trends, policies, characteristics and student generation. This model was then subjected to expert review.

### In-Depth Interviews with Experts

A panel of 10 faculty development experts from medical schools in Indonesia, with characteristics as presented in Table 2, participated in the interviews. Six experts were from public medical schools, and four were from private and religion-based medical schools. Seven of them represented schools from the Java region, where most medical schools reside; one was from Sumatra Island, one was from Sulawesi island and another was from the Nusa Tenggara region. The average time of an interview was 52 min.

The interviews resulted in four main themes with several subthemes (Table 3). The themes are factors that influence existing faculty development programmes and components of the faculty development model, its benefits and its challenges in implementing the model.

The results of the interviews showed that institutions have a significant role in implementing faculty development programmes. Some respondents stated that the implementation of faculty development is varied, influenced by the level of institutional awareness regarding the need for such development.

*Its [faculty development] implementation is now varied and individual, [and] depends on each institution and the level of awareness of the institution on the importance of faculty development*. (T1)

Respondents agreed that needs analysis is the main step in preparing faculty development programmes in Indonesia. Needs analysis is conducted to identify the needs of the faculty based on an evaluation of teaching practices and feedback from students or fellow teachers.

*Based on the needs of the faculty, we design our planning programmes… we also routinely evaluate the implementation of modules…, collect feedback from the students to identify their learning needs, as well as get feedback from the lecturers to find out their needs; [and] what materials need to be given to them*. (T7)

Respondents added that, so far, most of the implementation of faculty development programmes is still based on situational needs. One of the hurdles is the funding factor, which often makes faculty development programmes less than optimal.

*Proposals for such activities [faculty development] are still facing funding-related problems, such as …limited budgets and [there] are other activities that are more prioritised*. (T5)

Although a faculty development programme may be supported by an institution, it will not produce expected results if the participants are not motivated to be involved. The respondents described the low motivation of the faculty as one of the problems of faculty development implementation in Indonesia.

*So, it seems like… the policy already exists, the leaders or heads of the departments have supported the programme, but the awareness of the faculty is low so it feels like they do not really need it*. (T2)

Regarding the implementation of a faculty development model, respondents agreed that a large generation gap between students and teachers poses its own challenges in model implementation, especially in implementing technology-related concepts in the teaching process. Resistance to new concepts, although considered a normal response when something new is introduced, should also be identified and properly overcome. Another challenge in implementing the model is the difficulty of clinical faculty members to manage time between clinical practices, teaching and personal development.

Respondents also stated that local culture will affect how teachers interact with each other, and this will shape the institutional culture.

*Here, the bonds between teachers are strongly felt, maybe because we are all Minangnese. This way, we will especially pay attention to junior lecturers or new juniors, to a degree, when they bring new information. Junior lecturers will not hesitate to share their knowledge. Maybe it is one of the advantages of the culture here. In Java, especially in big cities, it is dominated by individualism, so it may affect the culture, in my opinion*. (T6)

During the interview process, the respondents were shown the proposed faculty development model in order to obtain their responses regarding it. According to the experts, the faculty development model is expected to describe the skills needed by teachers to develop their skills, not only in teaching but also skills in their areas of expertise. A faculty development programme is also expected to contribute to the attainment of the vision and mission of the institution.

The respondents proposed an ideal faculty development model that consists of three main components—content, process and system—that influence the implementation of a faculty development programme. Each component is further broken down, and Table 4 provides a comparison between the components of a faculty development model based on the literature review and interviews with experts.

**Figure 2:**
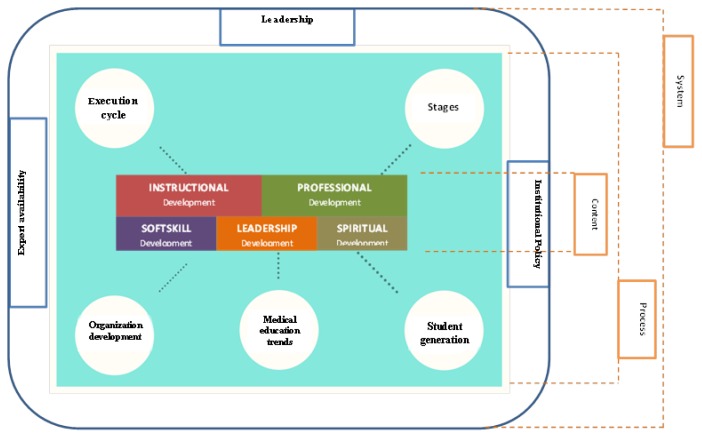
Final model of faculty development model based on literature and experts review

Combining the findings from the literature review and in-depth interviews, a final model of faculty development was formulated (Figure 2). The content component is every piece of content or material that needs to be delivered in a faculty development programme, including instructional development, professional development, soft skills, spiritual development and leadership skills. The process component consists of the implementation (execution) cycle, stages of faculty development, student generation, medical school trends and organisational development. Finally, the system component is the aspect of the educational system that affects the faculty development programme. The parts of this educational system are leadership, institutional policy and the availability of experts.

## Discussion

The model was first developed based on the findings from the literature review and further refined through interviews with faculty development experts. Results from in-depth interviews strengthened the proposed model because the interviews involved representative experts from various medical schools in Indonesia. The process of formulating a model consisting of these two stages has a distinct advantage compared to other faculty development models that used only one stage of development (27, 28, 29).

This study produced three main components in a faculty development model: content, process and system. These three components have made this model more comprehensive and ideal to be practiced in medical schools in developing countries, particularly Indonesia. The distinctive characteristics of this faculty development model are within the process and system components. Content-wise, almost no new faculty development materials arose from the expert review, except for spiritual development. The process and system components in this model have considered and accommodated the challenges and difficulties in developing countries. For example, the staging of a faculty development programme may help an institution set priority programmes based on limited funding and available teaching experts. The acknowledgement of the expert availability component in the faculty development model enables expert and resource sharing among medical schools in the region.

The content component covers all materials required in faculty development programmes in which instructional development skills are still the most significant domain (16, 17, 18, 21). Leadership development is also necessary for not only those in upper management but teachers as well, because they will need leadership skills to conduct their teaching tasks (28). The changes in learning paradigm undoubtedly should be followed with the improvement of soft skills, which benefits both the faculty and students (30).

Respondents in this study agree that the inclusion of spirituality in the faculty development model will benefit both students and faculty members by having regular spirituality-related discussions among faculty members or routine sermons/preaching to encourage spiritual development. In a religion-based institution, spiritual development is important in a faculty development programme (31). Moreover, Memaryan et al. (32) argued that incorporation of spirituality in the medical education curriculum is important to provide better care. However, there are challenges to this, such as the lack of agreement on what spirituality comprises and also the lack of training for staff to develop spirituality courses and to support students in aspects related to spirituality (33). A community’s culture and beliefs will also act either to promote or hinder spiritual development in that community (32).

The process component includes all aspects that influence implementation of the faculty development programme, from needs analysis to evaluation at the end of the programme, in all stages of the programme. The staging of a faculty development programme should consider aspects such as teaching experiences, teaching context and career path. Student generation and trends in medical education are also important topics to be discussed in the faculty development programme, especially to help teachers determine appropriate teaching approaches and content delivered to students (2, 24).

The system component, which highly correlates with the educational system of each institution, also plays an important role. Leadership is one aspect of the system, and it concerns how far leaders prioritise the faculty development programme and their understanding and commitment to faculty development (34). Institutional policies also have some influence, because they underline the programme, whether or not the institution considers faculty development to be important, or perhaps the institution is still focusing on completing basic requirements for running medical schools, such as curriculum, teaching resources and number of faculty members. In addition, the availability of experts affects the feasibility of implementing a faculty development programme (35).

The development and increased use of technology in medical education is inevitable (36), including in faculty development programmes. However, faculty members may not be comfortable or prepared to use and interact with technology. This is one challenge identified in the current study. Therefore, faculty development programmes need to be delivered using technology, such as webinars and online courses, to allow teachers to experience learning using technology for themselves. In their systematic review, Steinert et al. (37) found increased use of online learning as a viable instructional method in faculty development programmes.

Another finding in this study is the conflicting schedule and commitment of faculty members, which may hinder them from engaging in faculty development programmes. Therefore, early socialisation and implementation of this model is expected to benefit both medical schools and teachers. The balance between consistency and flexibility is important to make faculty development programmes accessible for all teachers, to encourage participation of all stakeholders and to maintain the quality and quantity of the planned programme (28).

Existing faculty development models originated from Western contexts, so there is a need to have a model that will suit the characteristics of medical schools in different contexts. As previously discussed, the improvement of educational quality is mostly affected by the quality of the institution’s faculty members (16). The experts interviewed in this study were the representatives of medical schools selected based on a representation of the regions in Indonesia, public and private institutions and religion-based institutions. Regional representation allows for the consideration of cultural differences in each medical school in Indonesia based on the representative types of institutions to help the researcher gain perspectives of different schools with different barriers and enablers. This faculty development model is feasible because it was designed based on various components that suit the characteristics and conditions of medical schools in Indonesia and very likely, other developing countries with similar characteristics.

According to Kim et al. (9), in most developing countries, increased access to higher education is not always followed by the improvement of the quality of the faculty members. Therefore, our model will help schools to stage a faculty development programme based on available funding, needs analysis results and teaching staff assignments. The list of contents in the model will also be useful for the faculty development leader to select the prioritised content for the particular school aligned with the vision and mission of the school. Clear stages of a faculty development programme for every teacher can be designed by considering teachers’ career pathways, and then adequately socialised. Teachers can also reflect on this model to identify the components required to become competent medical teachers.

The researchers acknowledge the limitation of the literature review, which may have missed models developed in languages other than English. However, a triangulation through in-depth interviews with representative experts has enabled the incorporation of different points of view.

## Conclusion

This faculty development model is produced through the comprehensive process of a literature review and expert interviews, which have made this model ideal and suitable for medical schools in Indonesia and other developing countries that share similar situations. This model, which consists of content, process and system components, is expected to assist medical schools in preparing a more comprehensive and sustainable faculty development programme that will lead to improved educational practices.

## Figures and Tables

**Table 1:** List of included articles and data extracted

Article	Definition of faculty development	Objectives of faculty development	Model/framework/guidelines used to design faculty development programme	Characteristics of participants in faculty development programme	Approaches used in faculty development programme	Content discussed in the faculty development programme	Challenges encountered	Output and evaluation of faculty development programme
Behar-Horenstein et al. (22)	✓	✓	×	✓	✓	✓	×	×
Smith and Hardinger (20)	×	×	×	✓	✓	✓	×	×
Phuong and McLean (21)	✓	✓	×	✓	×	×	✓	✓
Onyura et al. (8)	✓	✓	✓	×	×	×	✓	×
Purba (16)	✓	✓	✓	×	×	✓	×	×
Amin et al. (19)	✓	✓	×	✓	✓	✓	×	×
Daley et al. (17)	✓	✓	✓	✓	✓	×	✓	×
Kim et al. (23)	✓	✓	×	✓	✓	✓	✓	×
Walters et al. (24)	✓	✓	×	✓	✓	×	×	×
Hesketh et al. (18)	×	✓	✓	✓	×	✓	✓	✓

**Table 2:** Characteristics of the faculty development experts

ID	Status of the institution in which the expert works (public/private)	Years of working experience	Education level
T1	Public	9 years	Master
T2	Private	7 years	Master
T3	Public	18 years	PhD
T4	Public	16 years	Master
T5	Public	10 years	PhD candidate
T6	Public	15 years	Master
T7	Private	7 years	Master
T8	Public	12 years	Master
T9	Private	14 years	PhD
T10	Private	9 years	Master

**Table 3:** Themes and subthemes obtained from the expert interview

Theme	Sub-theme
Factors influencing existing faculty development programme	Institutional awareness
	Needs analysis
	Funding
	Motivation
Components of faculty development model	Content
	Process
	System
Benefits of faculty development model	Improving skills in teaching
	Improving content expertise
	Strengthening the institution’s vision and missions
Challenges in implementing faculty development model	Generation gap
	Resistance to new concepts
	Difficulty of faculty members to manage time
	Working/institutional culture

**Table 4:** Components of faculty development model

Components obtained from literature review	Components obtained from expert interview
Instructional skills development	Soft skills development (content)
Organisational skills development	Spiritual development (content)
Leadership skills development	Stages of faculty development (process)
Professional development	Implementation cycle (process)
Government/institution policy	Role of leaders (system)
Medical education trends	Expert availability (system)
Student generations	
Institutional characteristics	
